# Soil water consumption, water use efficiency and winter wheat production in response to nitrogen fertilizer and tillage

**DOI:** 10.7717/peerj.8892

**Published:** 2020-04-30

**Authors:** Shahbaz Khan, Sumera Anwar, Yu Shaobo, Zhiqiang Gao, Min Sun, M. Yasin Ashraf, Aixia Ren, Zhenping Yang

**Affiliations:** 1 College of Agriculture, Shanxi Agricultural University, Taigu, Shanxi, China; 2 Institute of Molecular Biology and Biotechnology, The University of Lahore, Lahore, Punjab, Pakistan

**Keywords:** *Triticum aestivum* L., Soil water content, Loess plateau, Nitrogen use efficiency, Protein yield, Tillage practices, WUE, Biomass, Grain yield, Bulk density

## Abstract

Sustainability of winter wheat yield under dryland conditions depends on improving soil water stored during fallow and its efficient use. A 3-year field experiment was conducted in Loess Plateau to access the effect of tillage and N (nitrogen) rates on soil water, N distribution and water- and nitrogen-use efficiency of winter wheat. Deep tillage (DT, 25–30 cm depth) and no-tillage (NT) were operated during fallow season, whereas four N rates (0, 90, 150 and 210 kg ha^−1^) were applied before sowing. Rates of N and variable rainfall during summer fallow period led to the difference of soil water storage. Soil water storage at anthesis and maturity was decreased with increasing N rate especially in the year with high precipitation (2014–2015). DT has increased the soil water storage at sowing, N content, numbers of spike, grain number, 1,000 grain weight, grain yield, and water and N use efficiency as compared to NT. Grain yield was significantly and positively related to soil water consumption at sowing to anthesis and anthesis to maturity, total plant N, and water-use efficiency. Our study implies that optimum N rate and deep tillage during the fallow season could improve dryland wheat production by balancing the water consumption and biomass production.

## Introduction

The world’s largest Loess Plateau is located in northern China, covering Shanxi, eastern Gansu, Shaanxi, and northern Henan provinces (Encyclopedia Britannica, 2013). Loess Plateau in China covers about 0.65 million km^2^ area and having 108 million population (Wang & Li, 2010). Loess Plateau has a semiarid climate with low and variable rainfall from 300–700 mm (Li & Xiao, 1992). Due to the lack of irrigation resources and deep and sparse groundwater, most of the agriculture is dryland farming completely depends on the precipitation (Zhang et al., 2009). Furthermore, there is a large inter-annual variation in precipitation, such as a wet year may receive two to five times more rainfall than the dry year. Therefore, the production of winter wheat and other crops in Loess Plateau varies greatly with the distribution pattern and rate of rainfall (He et al., 2014).

The dryland winter wheat (*Triticum aestivum* L.) is the most important crop of Loess Plateau with approximately 4.3 million hectares cultivating area (Wang et al., 2010). Winter wheat in this region is usually cultivated as a single crop per year followed by more than three months of summer bare fallow. Most of the precipitation (50–60%) in Loess Plateau falls in summer from June to September (Zhang et al., 2009). Therefore, precipitation stored in the soil during the summer fallow period after wheat harvest is utilized by the subsequent crop and crucial for the success of cropping in Loess Plateau (Zhang et al., 2007; Schlegel et al., 2017).

Water storage in soil has been affected by the different management practices such as tillage and fertilizer application (Grigoras et al., 2012). Winter wheat yield has been increased by the application of fertilizer but it also resulted in increasing soil water depletion and formation of dry subsoil layer especially in the high land areas (Yan et al., 2015). Hence, for sustainable wheat production, it is crucial to seek management practices for improving water-and N-use efficiency (Zhang et al., 2009; Fu et al., 2014). In rain-fed agriculture, tillage operation during the summer fallow period increases the rainfall infiltration and soil water storage (Wang et al., 2016). Although the proper use of tillage practices overcomes edaphic constraints, inappropriate and extensive tillage may destroy soil structure, accelerated erosion, loss of organic matter and soil fertility, and disruption of water and nutrient cycle (Lal, 1993).

Therefore, to enhance the sustainability and controlling erosion, arable farmers are shifting from traditional tillage to conservation tillage with minimum or no-tillage (Lal, Reicosky & Hanson, 2007; Kassam et al., 2009). No-tillage may significantly reduce soil erosion by eliminating plowing and improved other soil properties, reduced the bulk density, and increased organic matter, porosity, available water content and root mass density (Wang et al., 2010; Hansen et al., 2012; Alam et al., 2014).

However, long term practice of no-tillage and shallow tillage has resulted in the formation of hardpan and subsoil compaction, which restrict root penetration and reduce water and nutrient uptake from deep layers, thus reducing drought resistance of wheat crop with a yield reduction in most parts of northern China (Bengough et al., 2011; Zhang et al., 2018). Furthermore, the continuous adoption of no-tillage resulted in a progressive decrease in grain yield compared with tillage, mainly attributable to a decrease in soil N availability (Amato et al., 2013; Ruisi et al., 2016). Some authors described that tillage increases N availability by stimulating microbial activity in the soil (Soon et al., 2007; Ruisi et al., 2016). Deep tillage is a suitable strategy to break the hardpans in deeper soil which increase the porosity of the soil and homogeneously distribute the soil moisture (Tapanarova, 2005; Ahmad et al., 2010).

Nitrogen (N) is the most important nutrient for ensuring both high grain yield and grain quality (Khan et al., 2017). But the heavy application of N fertilizer represents a significant cost and also cause serious environmental problems due to the loss of a large amount of applied N into the environment (Ma et al., 2019). Optimizing the input of N is difficult under rain-fed cropping system due to the highly variable weather and rainfall. Less N supply may limit grain yield and grain protein content and excessive application of N may increase water use in the early growing season leading to water deficit stress during flowering and grain filling, resulting in poor grain set (He et al., 2014). At the same time, tillage practices also have a significant influence on N leaching (Huang et al., 2015). Optimum N application in combination with proper tillage practice is expected to sustain soil fertility and wheat yield while decreasing N leaching (Zhou & Butterbach-Bahl, 2014). Grain protein is determined by the balance between the N requirement of a crop and the supply of N, as well as by water availability and environmental conditions during grain filling. Yield and grain protein concentration are often negatively correlated (Ma et al., 2019).

Evaluating the optimal N fertilizer rate and soil water balance for increasing N and water use efficiency are important issues in water-saving agriculture (Fu et al., 2014). Although some previous research indicated that the winter wheat production of Loess Plateau could be substantially increased by optimizing the N input rate (Fu et al., 2014; Ren et al., 2019; Zhong & Shangguan, 2014) and adopting deep tillage practices which improve the soil bulk density and precipitation use efficiency (Jin et al., 2007; Sang, Wang & Lin, 2016; Xue et al., 2019). However, the N input rates have not been previously optimized according to the precipitation distribution mainly the fellow season precipitation under different tillage to properly utilize the soil water content. Therefore, our main objective was to explore the interactive effect of different N rates and tillage (deep tillage and no-tillage) on soil water consumption, biomass production and N allocation and to optimize N rate according to the precipitation rate.

## Materials & Methods

### Description of experimental site

Field experiment was performed at the at the Agriculture Research Station of Shanxi Agriculture University (35°20′N, 111°17′E), in Wenxi county, Shanxi Province of China. The experimental site was located in the southeast of the Loess Plateau. This region is characterized by semiarid climate which receives 491 mm of average annual precipitation, 12.9 °C annual mean temperature, and 2242 h of annual sunshine.

### Field management and experimental design

The experiment, a typical winter wheat-summer fallow, was started with the winter wheat season of October 2014, and ended after the winter wheat was harvested in June 2017, covering 3 successive wheat crops at the same experimental plot.

**Figure 1:**
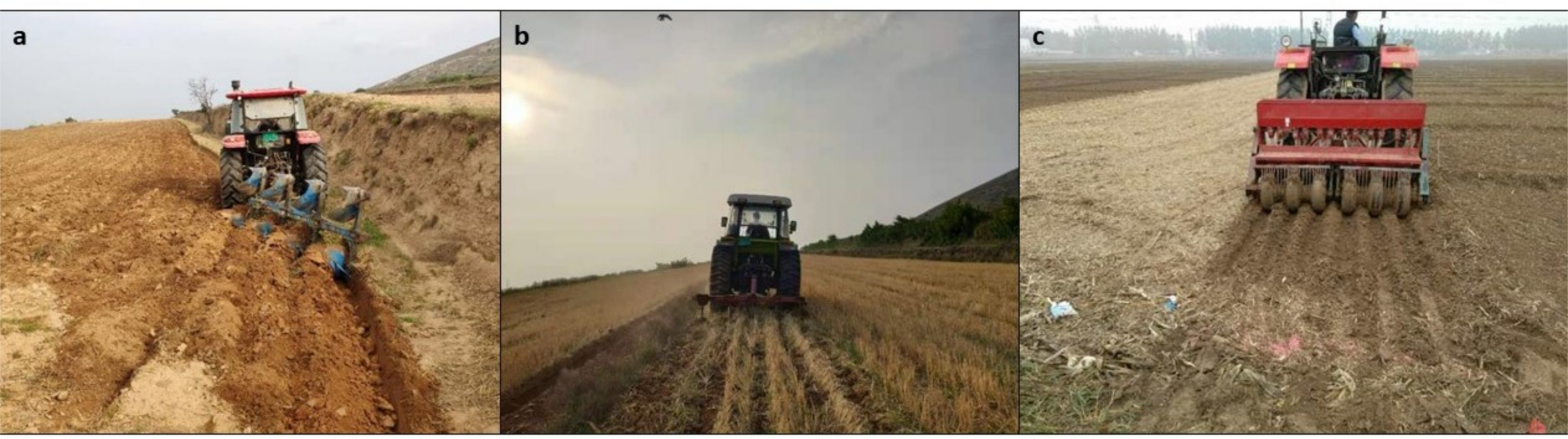
Different tillage practices performed during the field preparation: (A) deep tillage, (B) subsoiling, and (C) no-tillage.

**Figure 2:**
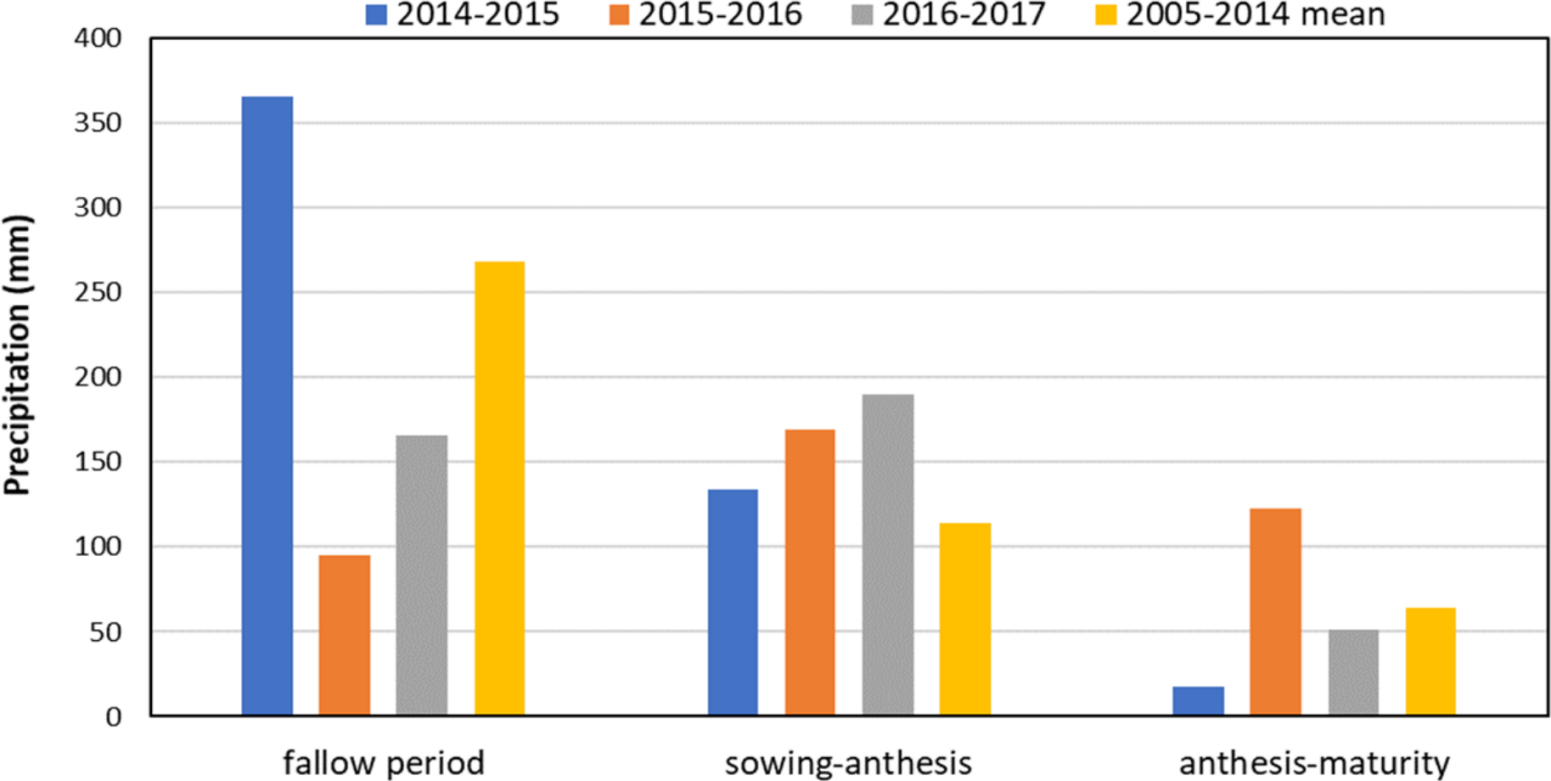
Precipitation distribution during study years (2014–2017) and average precipitation in 2005–2014 in different growth stages of wheat at the experimental site in Wenxi, China.

The seeds of winter wheat (*Triticum aestivum* L.) cultivar ‘Yunhan 20410’ were obtained from Wenxi Agriculture Bureau. Wheat stubble of 20–30 cm from the previous wheat crop was left in field to reduce evaporation and to increase organic carbon content in soil. The experiment was set as split-plot design with RCBD (randomized complete block design). The whole experimental field was divided into 2 main plots i.e., tillage methods (no tillage and deep tillage) and each main plot was divided into 4 subplots and levels of nitrogen were randomized over the subplots. Each treatment was repeated 3 times. In mid-July, deep tillage (DT) was operated with furrow plough (TH-FZL-2, Tianhe machinery equipment factory, Jinan, China) at the depth of 25–30 cm during fallow season, whereas no-tillage (NT) was taken as a control (Fig. 1). Each main plot was divided into four sub-plots and different N rates (0, 90, 150 and 210 kg ha^−1^) were applied as urea fertilizer (46% N). The area of each sub-plot was 30 m^2^ (3 m wide and 10 m long). N fertilizer was applied in a single dose before sowing and no additional fertilizer was applied during the growth period. The description of experimental setup is given in Table 1. In late August, rotary tillage was performed at a depth of 12–15 cm to crumble large lumps and to properly incorporate N fertilizer in soil. In October of each year from 2014 to 2017, seeds of winter wheat were sown by hand-drill at a seeding rate of 225 × 10^4^ seeds ha^−1^ and 20 cm row spacing. The field was remained fallow during each year from mid-June to September. The precipitation rate during fallow period and from sowing to anthesis and anthesis to maturity stage of winter wheat were recorded and presented in Fig. 2. At maturity, plants were harvested on 12 June 2015, 10 June 2016 and 3 June 2017.

**Table 1:** Description of tillage practice and nitrogen treatments.

Tillage	Nitrogen	Fertilizer	Practice during fallow season	Farming method	Cultivation method
No-Tillage (NT)	0, 90, 150 and 210 kg ha^−1^	N (urea containing 46% N), was applied in two split doses; P_2_O_5_ (150 kg ha^−1^), and K_2_O (90 kg ha^−1^) were applied once before sowing	No-tillage	rotary tillage and land leveling were performed before sowing for planting preparation	Seeds were sown at a density of 225 ×10^4^ ha^−1^ by drilling method and row spacing was 20 cm
Deep Tillage (DT)	0, 90, 150 and 210 kg ha^−1^	N (urea containing 46% N) was applied in two split doses; P_2_O_5_ (150 kg ha^−1^), and K_2_O (90 kg ha^−1^) were applied once before sowing	Deep tillage was performed with furrow plough at the depth of 25–30 cm	rotary tillage and land leveling were performed before sowing for planting preparation	Seeds were sown at a density of 225 ×10^4^ ha^−1^ by drilling method and row spacing was 20 cm

### Nitrogen accumulation and grain protein

At maturity stage, twenty plants were randomly collected from each treatment for the measurement of N contents in stem+sheath, glume+spike, grain and total plant N content. The total N concentration of the oven-dried, ground, and acid-digested plant samples was determined using the indophenol-blue colorimetric method (Meyer, 1983). Grain protein content was calculated as grain N content multiplied by 5.7.

### Nitrogen use efficiency

Apparent recovery nitrogen use efficiency (ARNUE), i.e., N uptake by plant (kg ha^−1^) per kg N applied was calculated as suggested by Khan et al. (2017).

(1) \begin{eqnarray*}\mathrm{ARNUE}=[({\mathrm{NU}}_{\mathrm{fi}}-{\mathrm{NU}}_{\mathrm{f0}})/\mathrm{N}]\times 100\end{eqnarray*}

where, NU_fi_ and NU_f0_ indicated N uptake of fertilized and unfertilized plants (kg ha^−1^) respectively, and N was the rate of N fertilizer (kg ha^−1^).

### Soil water balance

The soil samples were excavated after every 20 cm depths from 3 m soil depth using cutting ring method (Dam et al., 2005). Soil samples were collected from each subplot at the time of wheat sowing, anthesis and maturity. The soil water storage (SWS) of each soil layer was calculated using equation (Liu et al., 2016):

(2) \begin{eqnarray*}\mathrm{SWS}(\mathrm{mm})=\mathrm{BD}/\mathrm{\rho }\mathrm{w}\times \mathrm{SWC}\times \mathrm{D}\end{eqnarray*}

where BD is bulk density (g dry soil cm^−3^); ρw is water density (1 g cm^−3^); SWC is the soil water content (g water g^−1^ dry soil); and D is the depth of the soil profile (mm). The SWC and BD of each soil layer were determined by oven-drying method (Gardner, 1986).

In addition, soil water consumption from sowing to anthesis was calculated as the difference in SWS at sowing minus the SWS at anthesis. Soil water consumption from anthesis to maturity was calculated as the difference in SWS at anthesis minus the SWS at maturity.

Evapotranspiration (ET, mm) was calculated according to Ma et al. (2015) and He et al. (2016) as follows:

(3) \begin{eqnarray*}\mathrm{ET}=\mathrm{P}-\Delta \mathrm{SWS}\end{eqnarray*}

where P (mm) is the amount of precipitation at the site as shown in Fig. 2, and ΔSWS (mm) is the changes in soil water storage due to water consumption and was measured as the difference of soil water storage in the 0–300 cm soil profile at the sowing and harvesting stage of wheat.

### Water use efficiency

Water use efficiency (WUE, kg ha^−1^ mm^−1^) was quantified by dividing grain yield with evapotranspiration as follow:

(4) \begin{eqnarray*}\mathrm{WUE}=\mathrm{GY }/\mathrm{ET}\end{eqnarray*}

ET is evapotranspiration (mm) calculated from Eq. (3) and GY is the grain yield (kg ha^−1^)

### Grain yield, grain protein yield

At maturity, plants were randomly sampled from three 1 m^2^ areas from each plot to determine grain number spike^−1^ and 1,000 grain weight. All plants from the plots were harvested on 12 June 2015, 10 June 2016 and 3 June 2017. Grains were air-dried whereas aboveground plant parts were oven dried until constant weight to determine the grain yield (kg ha^−1^) and dry biomass. The harvest index (HI) was calculated dividing the grain yield by the aboveground dry biomass.

### Statistical analysis

Three-way analysis of variance was conducted using SAS 8.1 (SAS Corp., Cary, NC, USA) to analyze the significance of tillage, nitrogen treatments (N), and their interaction. When F-values were significant, the least significant difference (LSD) test was used to compare means. A linear regression analysis was performed to predict the grain yield and grain protein yield with soil water consumption. Graphs were drawn using Microsoft Excel 2010 (Microsoft Corp., Redmond, WA, USA). Pearson correlation coefficients were assessed at *P* < 0.05 using R statistical software (version 3.2.3).

## Results

### Precipitation distribution

The annual precipitation in 2014–2015 was 70.2 mm higher than the average long-term precipitation at the site (2005–2014), whereas, annual precipitation during 2015–2016 and 2016–2017 was 59.7 mm and 40.2 mm less than the average long-term precipitation (Fig. 2). In 2014–2015, most of the precipitation occurred during the fallow season with 97.2 mm higher than the long-term precipitation. In 2015–2016, fallow season precipitation was less whereas, precipitation from sowing-anthesis and anthesis-maturity were higher than the long-term precipitation.

### Effects of tillage on soil properties

Table 2 showed the soil properties from the field which received DT and NT. The organic content, available P and available N in 0–20 cm soil depth were increased in DT compared to NT during all three years, whereas, bulk density of 0–20 cm and 20–40 cm soil depth was significantly decreased by DT. By the application of DT, 6 and 19% reduction in bulk density was recorded before sowing and 7 and 15% reduction after the harvest of wheat in 0–20 cm and 20–40 cm soil depth as compared to NT.

**Table 2:** Effect of different tillage treatments on soil organic carbon, available phosphorus, available nitrogen and bulk density.

Soil properties	period	depth	Year	tillage	
Soil organic carbon (g kg^−1^)	before sowing	0–20 cm	2014/15	NT	8.57 a
						DT	8.71 a
					2015/16	NT	8.82 a
						DT	8.91 a
					2016/17	NT	8.72 a
			DT	8.75 a	
Available phosphorus (mg kg^−1^)	before sowing	0–20 cm	2014/15	NT	11.26 b
						DT	12.83 a
					2015/16	NT	9.94 b
						DT	13.06 a
					2016/17	NT	13.01 a
			DT	13.56 a	
Available nitrogen (mg kg^−1^)	before sowing	0–20 cm	2014/15	NT	31.00 b
						DT	36.25 a
					2015/16	NT	46.03 b
						DT	66.03 a
					2016/17	NT	30.99 b
			DT	36.41 a	
Bulk density (g/cm^3^)	before sowing	0–20 cm	2015/16	NT	1.31 a
							DT	1.23 b
					20–40 cm		NT	1.49 a
						DT	1.20 b
		harvest		0–20 cm		NT	1.40 a
							DT	1.30 b
					20–40 cm		NT	1.68 a
				DT	1.43 b

**Notes.**

DT deep tillage NT no-tillage

Different letters in the column indicate significant difference among treatments at *p* < 0.05 by the LSD test.

### Effects of tillage on soil water storage

**Figure 3:**
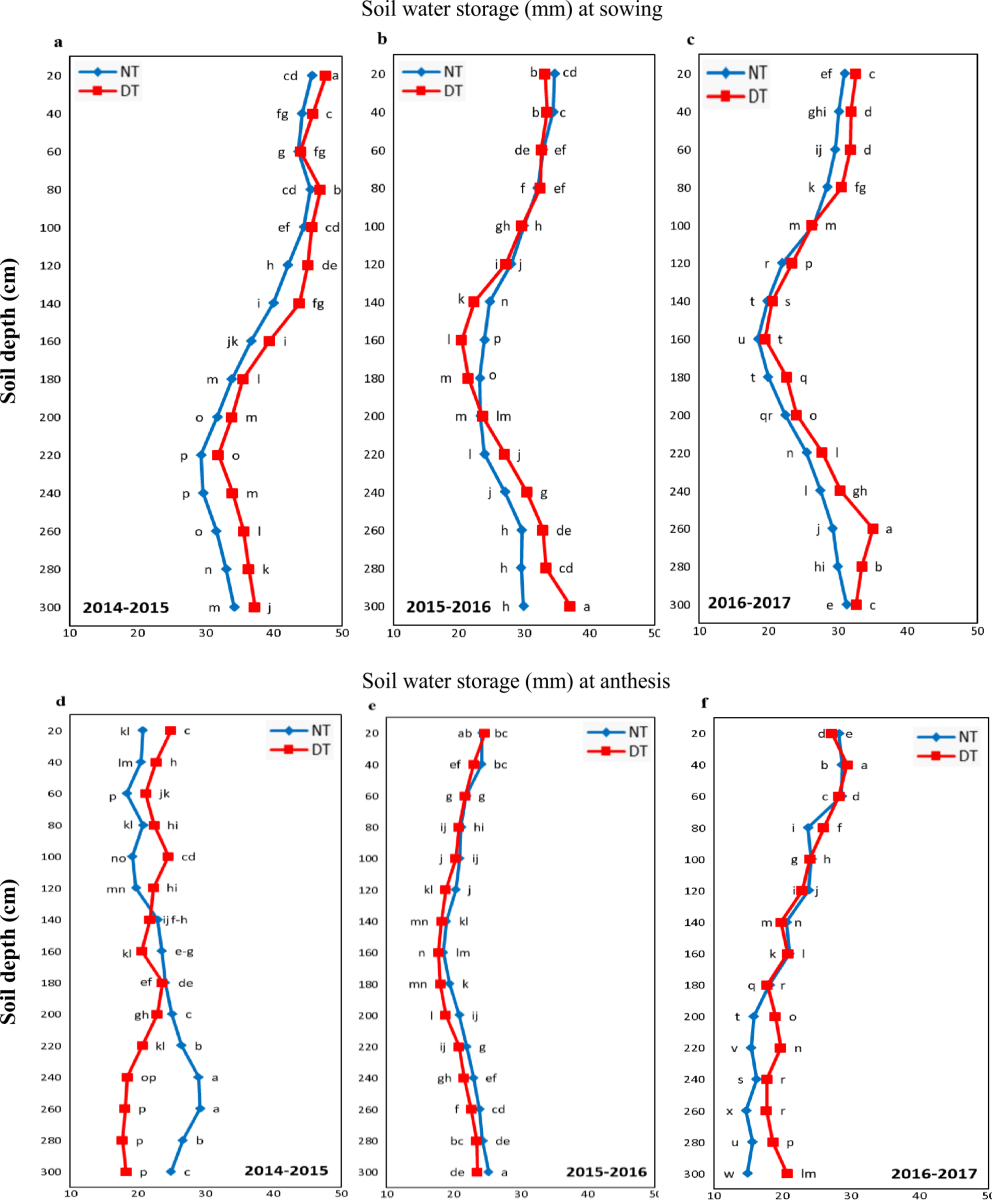
Soil water storage at 0-300 cm soil depth under no-tillage (NT) and deep tillage (DT). (A, B & C) Soil water storage at sowing, (D, E & F) Soil water storage at anthesis of winter wheat. Different letters indicated significant differences among treatments by Fisher’s LSD test.

The soil water storage also varied with years (Fig. 3). In 2014-2015, the precipitation during fallow season and the soil water storage at sowing and anthesis was higher than in 2015–2016 and 2016–2017. Soil water storage in 0–300 cm at sowing was increased with DT as compared to NT. At sowing, average soil water storage across all years in 0–300 cm soil layers was 461.7 mm under NT and 487.6 mm in DT. The effect of DT was less prominent in 2015–2016 with high precipitation at sowing. Water content at 20–40 cm and 80–300 cm in 2014–2015, 220–300 cm in 2015–2016, and 20–80 cm and 180–300 cm in 2016–2017 were higher under DT than NT. Soil water storage at anthesis was less under DT than NT in 2014-15 and 2015–2016, whereas, in 2016–2017, soil water storage at DT was higher than NT.

### Effect of N rates on soil water storage and rain water use

The N rate was applied at the time of sowing therefore, the soil water storage before sowing was not affected by the N rate (Table 3). Soil water storage at anthesis and maturity was decreased with increasing N rate and a more significant decrease was observed in the year with high precipitation (2014–2015). In 2014–2015 and 2016–2017, soil water storage was highest at sowing, followed by at anthesis and then at maturity, whereas in 2015–2016, soil water storage at maturity was higher than at anthesis.

**Table 3:** Soil water storage (0–300 cm soil layer) at sowing, anthesis and maturity of winter wheat under different *N* levels.

Years	N rate	Soil water storage (mm)		
		before sowing	anthesis	maturity
2014/15	0	600.3 a	435.1 b	374.1 e
	90	600.3 a	416.8 bc	354.6 ef
	150	600.3 a	393.3 cd	328.3 fg
	210	600.3 a	376.7 d	319.5 g
2015/2016	0	414.6 a	285.1 c	319.1 bc
	90	414.6 a	280.5 c	316.0 b
	150	414.6 a	262.3 c	312.5 c
	210	414.6 a	260.5 c	302.7 b
2016/2017	0	412.7 a	332.7 b	304.5 d
	90	412.7 a	319.2 b	293.8 d
	150	412.7 a	329.8 b	283.5 d
	210	412.7 a	323.3 b	295.4 d
ANOVA (*F*-values)	Years (Y)	2315.8^∗∗∗^	900.4^∗∗∗^	117.3^∗∗∗^
		Nitrogen (N)	0.00^ns^	26.56^∗∗∗^	21.54^∗∗∗^
	Y × N	0.00^ns^	8.08^∗∗∗^	6.47^∗∗∗^

**Notes.**

*, **, *** significant at 0.05, 0.01 and 0.001 probability level, respectively. Different letters indicate significant differences (*p* ≤ 0.05) among treatments by least significant difference.

**Figure 4:**
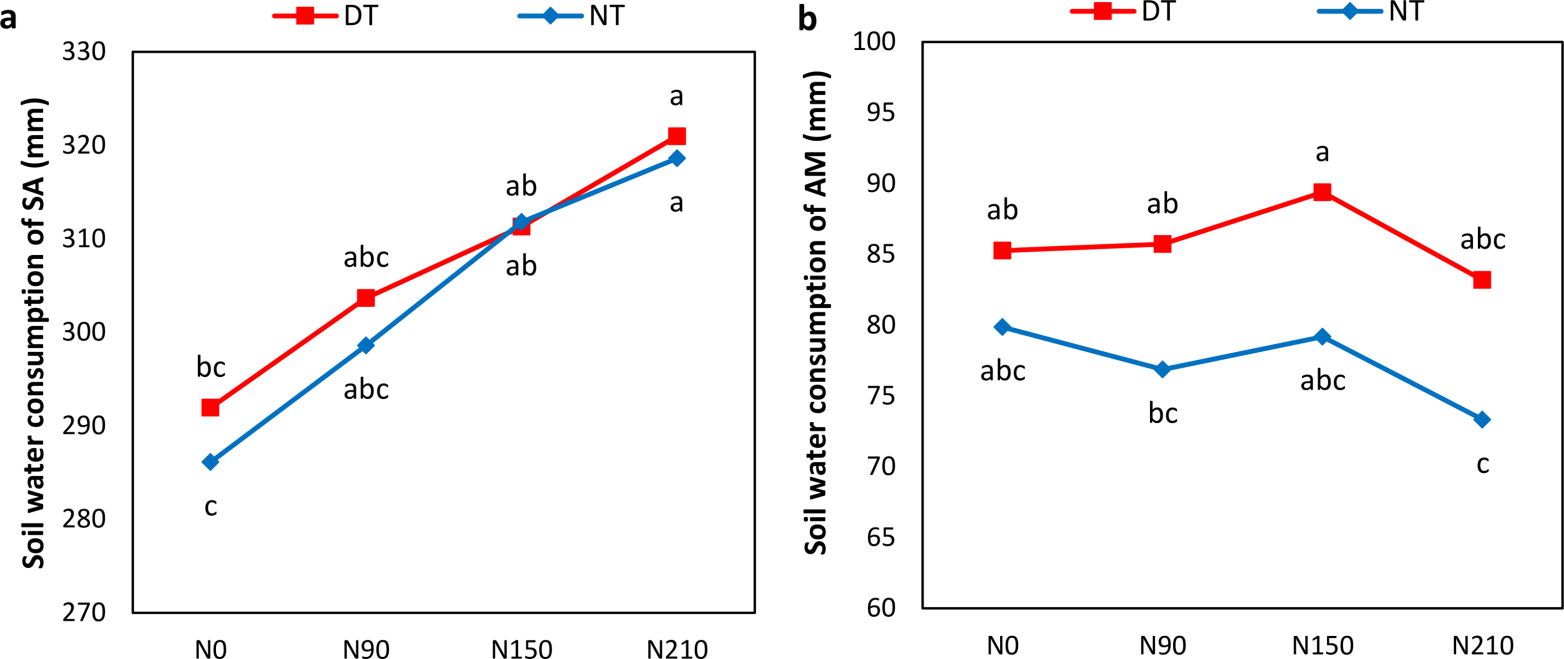
Effect of different nitrogen levels under deep tillage and no-tillage on soil water consumption of winter wheat. (A) Soil water consumption from sowing to anthesis, and (B) soil water consumption from anthesis to maturity of winter wheat. (NT, no-tillage; DT, deep tillage; SA, sowing to anthesis; AM, anthesis to maturity; N0, N90, N150, and N210 indicated 0, 90, 150 and 210 kg N ha^−1^). Different letters indicate significant differences (*p* < 0.05) among treatments by Fisher’s least significant difference.

Soil water consumption from sowing to anthesis (SA) was linearly increased by increasing N rates with the highest consumption recorded at 210 kg ha^−1^ (Fig. 4). Soil water consumption from anthesis to maturity (AM) was highest at 150 kg ha^−1^. Soil water of SA was affected more than at AM.

### Effect of tillage and N rate on N allocation in aboveground plant parts, yield traits, plant biomass, WUE, and NUE

The allocation of N in plant parts was significantly affected by the year, tillage, and N rates (Table 4). N accumulation in all plant parts was increased by increasing N rate from 90 kg ha^−1^ to 150 kg ha^−1^ both under DT and NT. N accumulation was decreased by further increasing N rate to 210 kg ha^−1^. Allocation of N to aboveground plant parts was higher under DT than NT. Maximum N content of leaf, stem+sheath, glume+spike and grain was recorded under DT at 150 kg N ha^−1^, except for the N content in stem+sheath in 2014/15 which was highest at the same concentration under NT. Minimum N content in leaf, stem+sheath, grain and total plant N was found under NT without N. The effect of interaction among year, tillage and N rates was significant for N content in leaf, stem+sheath and glume+spike whereas non-significant for N content in grains and total plant N.

**Table 4:** The effect of different N treatments under deep tillage and no-tillage condition on N contents in plant parts of winter wheat in 2014 to 2017.

Treatments			N content (kg ha^−1^)				
Years	Tillage	Nitrogen	Leaf	Stem+sheath	Glume+spike	Grain	Total plant
2014/15	DT	0	3.59 de	12.99 cd	5.94 e	119.11 e	141.68 e
		90	3.86 d	12.66 de	8.55 a	131.26 bc	155.47 cd
		150	7.51 a	14.23 b	8.27 b	147.86 a	180.63 a
		210	5.39 b	13.59 bc	6.66 d	133.58 b	162.66 bc
	NT	0	3.28 e	11.12 f	4.81 f	108.79 f	132.97 f
		90	4.54 c	12.74 de	6.63 d	123.66 de	144.37 e
		150	4.77 c	16.82 a	7.49 c	134.46 b	164.40 b
		210	3.81 d	12.32 e	8.23 b	127.36 cd	154.08 d
2015/16	DT	0	2.89 e	10.54 de	3.49 e	72.92 c	89.84 d
		90	3.85 c	16.10 c	6.66 c	76.26 c	102.87 c
		150	5.64 a	21.85 a	8.87 a	98.88 a	135.24 a
		210	4.62 b	18.15 b	7.49 b	83.62 b	113.88 b
	NT	0	2.65 e	8.66 f	3.96 e	60.38 d	75.65 e
		90	3.33 d	11.77 d	5.03 d	60.59 d	80.72 e
		150	4.08 c	15.19 c	4.74 d	84.75 b	108.76 bc
		210	2.75 e	10.12 e	2.73 f	65.44 d	81.04 e
2016/17	DT	0	2.62 de	11.82 d	4.16 de	92.51 f	111.11 e
		90	3.26 cd	15.23 c	6.30 c	104.27 d	129.06 d
		150	5.77 a	22.77 a	9.77 a	130.89 a	169.20 a
		210	3.74 c	15.02 c	6.39 c	110.11 c	135.26 c
	NT	0	2.10 e	9.23 e	3.19 f	83.41 g	97.93 f
		90	2.65 de	11.37 d	4.67 d	92.85 ef	111.54 e
		150	4.63 b	16.85 b	7.71 b	115.16 b	144.35 b
		210	2.66 de	11.70 d	3.87 e	96.10 e	114.33 e
ANOVA (*F*-values)	Years (Y)		76.3^∗∗∗^	15.3^∗∗∗^	172.0^∗∗∗^	2172.6^∗∗∗^	1348.8^∗∗∗^
		Tillage (T)		143.2^∗∗∗^	446.5^∗∗∗^	427.9^∗∗∗^	355.4^∗∗∗^	420.5^∗∗∗^
		Nitrogen (N)		179.5^∗∗∗^	405.4^∗∗∗^	288.3^∗∗∗^	347.9^∗∗∗^	404.6^∗∗∗^
	Y × T × N		5.82^∗∗∗^	18.2^∗∗∗^	52.2^∗∗∗^	1.58^ns^	1.81^ns^

**Notes.**

Values are the means of three replicates. *, **, *** significant at 0.05, 0.01 and 0.001 probability level, respectively. Different letters in the column indicate significant difference among treatments at *p* < 0.05 by the LSD test.

Deep tillage has significantly increased the numbers of spike, grain number spike^−1^, 1,000 grain weight, and grain yield (Table 5). The maximum number of spikes in all years were observed at 150 kg N ha^−1^under DT. The maximum number of grains, 1,000 grain weight and grain yield were observed at 150 kg N ha^−1^under DT in 2015–2016 and 2016–2017, whereas in 2014–2015, maximum values of these values were obtained at 210 kg N ha^−1^ under DT. The maximum HI was recorded at 210 kg N ha^−1^ in 2014–2015 and 2015–2016, while in 2016–2017, HI was highest at 150 kg N ha^−1^. Grain protein content was increased linearly by the application of N from 0 to 150 kg ha^−1^ in all years. Application of 150 kg N ha^−1^ resulted in the maximum protein contents under NT and DT. The interaction among year, tillage and N rate was significant for all yield traits except for the spike numbers.

**Table 5:** Yield components of winter wheat grown under deep tillage and no-tillage condition and different *N* treatments from 2014 to 2017.

Treatments			Spike no. (10^4^ ha^−1^)	Grain no. (spike^−1^)	1,000 grain weight (g)	GY (t ha^−1^)	GP (%)	Harvest index
Years	Tillage	Nitrogen						
2014/15	DT	0	476.0 bc	31.38 e	40.52 ab	4.97 d	10.78 h	0.343 c
		90	482.2 abc	31.69 d	39.65 d	5.06 c	11.68 f	0.350 c
		150	506.0 a	32.61 b	40.07 c	5.33 b	13.61 b	0.363 ab
		210	493.7 ab	33.22 a	40.78 a	5.78 a	12.28 d	0.368 a
	NT	0	446.3 d	31.06 f	39.65 d	4.59 f	11.36 g	0.351 bc
		90	462.0 cd	30.94 f	38.86 e	4.93 de	11.97 e	0.374 a
		150	481.5 abc	32.25 c	39.71 d	4.89 e	13.96 a	0.364 ab
		210	476.2 bc	32.54 b	40.33 bc	5.36 b	12.98 c	0.369 a
2015/16	DT	0	464.2 de	31.20 bc	42.07 b	4.43 c	11.93 h	0.394 bc
		90	481.7 bcd	31.53 ab	40.93 d	4.48 c	12.44 f	0.348 e
		150	526.3 a	31.75 a	43.42 a	5.14 a	13.67 c	0.359 de
		210	498.5 b	29.06 e	40.74 d	4.71 b	13.26 d	0.373 cd
	NT	0	445.2 e	27.27 f	38.42 f	3.89 e	12.37 g	0.398 b
		90	464.8 de	25.12 g	38.63 f	3.99 e	12.98 e	0.364 de
		150	488.3 bc	30.90 cd	41.48 c	4.70 b	14.64 a	0.383 bcd
		210	476.8 cd	30.50 d	39.26 e	4.28 d	14.08 b	0.447 a
2016/17	DT	0	470.1 cd	31.10 de	40.51 b	4.57 d	13.42 c	0.344 b
		90	481.9 bcd	31.37 cd	40.52 b	4.88 c	13.49 c	0.340 b
		150	516.2 a	32.09 a	42.05 a	5.57 a	14.04 b	0.370 a
		210	496.1 ab	31.59 bc	40.86 b	4.99 b	14.38 a	0.339 b
	NT	0	445.7 e	30.90 e	38.57 d	4.15 f	13.56 c	0.344 b
		90	463.4 de	31.02 e	39.16 c	4.42 e	13.65 c	0.336 b
		150	484.9 bc	31.87 ab	41.91 a	5.08 b	14.37 a	0.361 a
		210	476.5 bcd	30.43 f	39.51 c	4.46 de	14.11 b	0.304 c
ANOVA (*F*-values)	Years (Y)		0.29^ns^	854.6^∗∗∗^	55.51^∗∗∗^	633.7^∗∗∗^	2157.4^∗∗∗^	102.6^∗∗∗^
		Tillage (T)		63.86^∗∗∗^	606.4^∗∗∗^	686.9^∗∗∗^	814.1^∗∗∗^	348.8^∗∗∗^	13.88^∗∗∗^
		Nitrogen (N)		38.87^∗∗∗^	255.1^∗∗∗^	223.4^∗∗∗^	409.1^∗∗∗^	1764.8^∗∗∗^	8.76^∗∗∗^
	Y ×T ×N		0.26^ns^	166.2^∗∗∗^	7.37^∗∗∗^	3.56^∗∗^	9.17^∗∗∗^	9.74^∗∗∗^

**Notes.**

GY grain yield GP grain protein content

Values are the means of three replicates; *, **, *** significant at 0.05, 0.01 and 0.001 probability level, respectively. Different letters in the column indicate significant difference among treatments at *p* < 0.05 by the LSD test.

**Figure 5:**
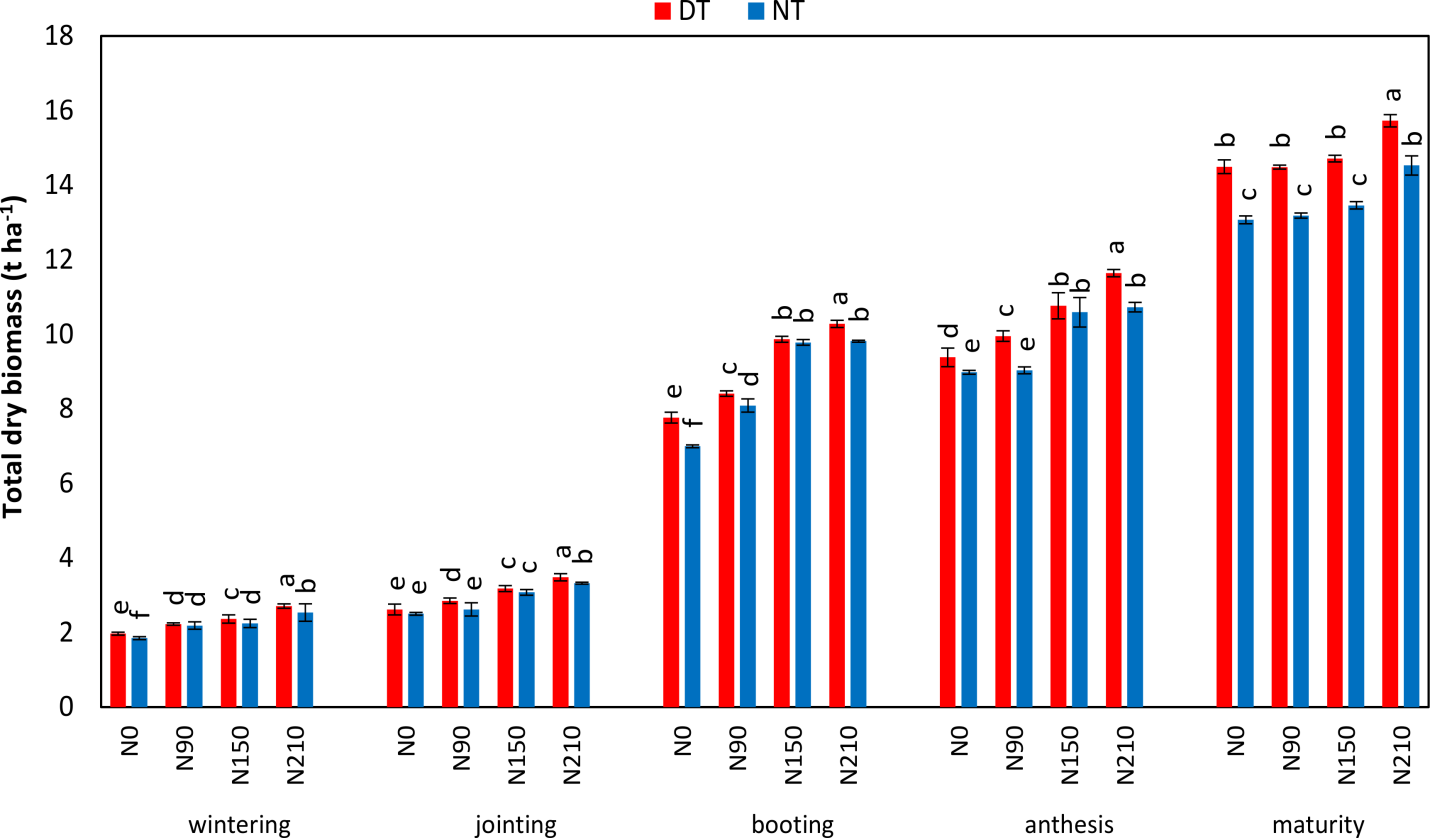
Effect of tillage practices and nitrogen levels on total plant biomass at different growth stages of winter wheat in 2014–2015. (NT, no tillage; DT, deep tillage; N0, N90, N150 and N210 indicated 0, 90, 150 and 210 kg N ha^−1^). Different letters indicate significant differences (*p* < 0.05) among treatments within a growth stage by Fisher’s least significant difference.

**Figure 6:**
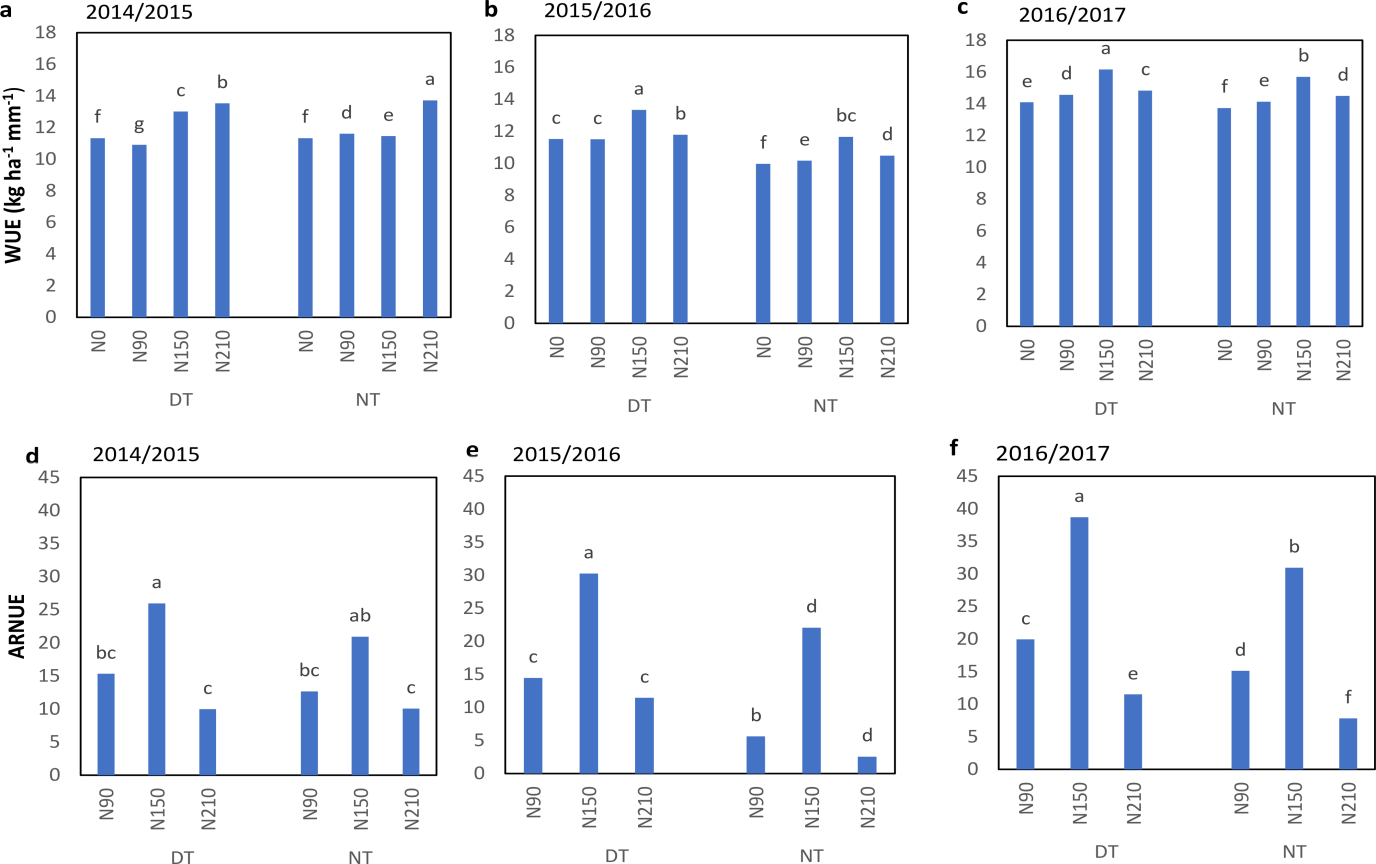
Effect of tillage treatments and N levels on WUE and ARNUE of winter wheat. (A, B & C) WUE and (D, E & F) ARNUE, (DT, deep tillage; NT, no-tillage; N90, N150, N210 indicated 90, 150 and 210 kg N ha^−1^). Different letters indicate significant differences (*p* < 0.05) among treatments by Fisher’s least significant difference.

Total dry biomass of plants under DT was higher than NT (Fig. 5). Increasing N rate significantly increased the biomass. Therefore, the highest biomass was recorded at 210 kg ha^−1^ under DT and minimum at NT without N. In 2014-2015, WUE was improved with increasing the N rate and highest WUE was found at 210 kg ha^−1^ (Fig. 6). Maximum WUE in 2015–2016 and 2016–2017 was recorded at 150 kg ha^−1^ under both NT and DT. NUE was significantly higher at 150 kg ha^−1^ than at 90 kg ha^−1^ and 210 kg ha^−1^. Overall WUE and NUE at DT was higher than NT.

### Relationship of grain yield and grain protein yield with soil water consumption, N content and other traits

**Figure 7:**
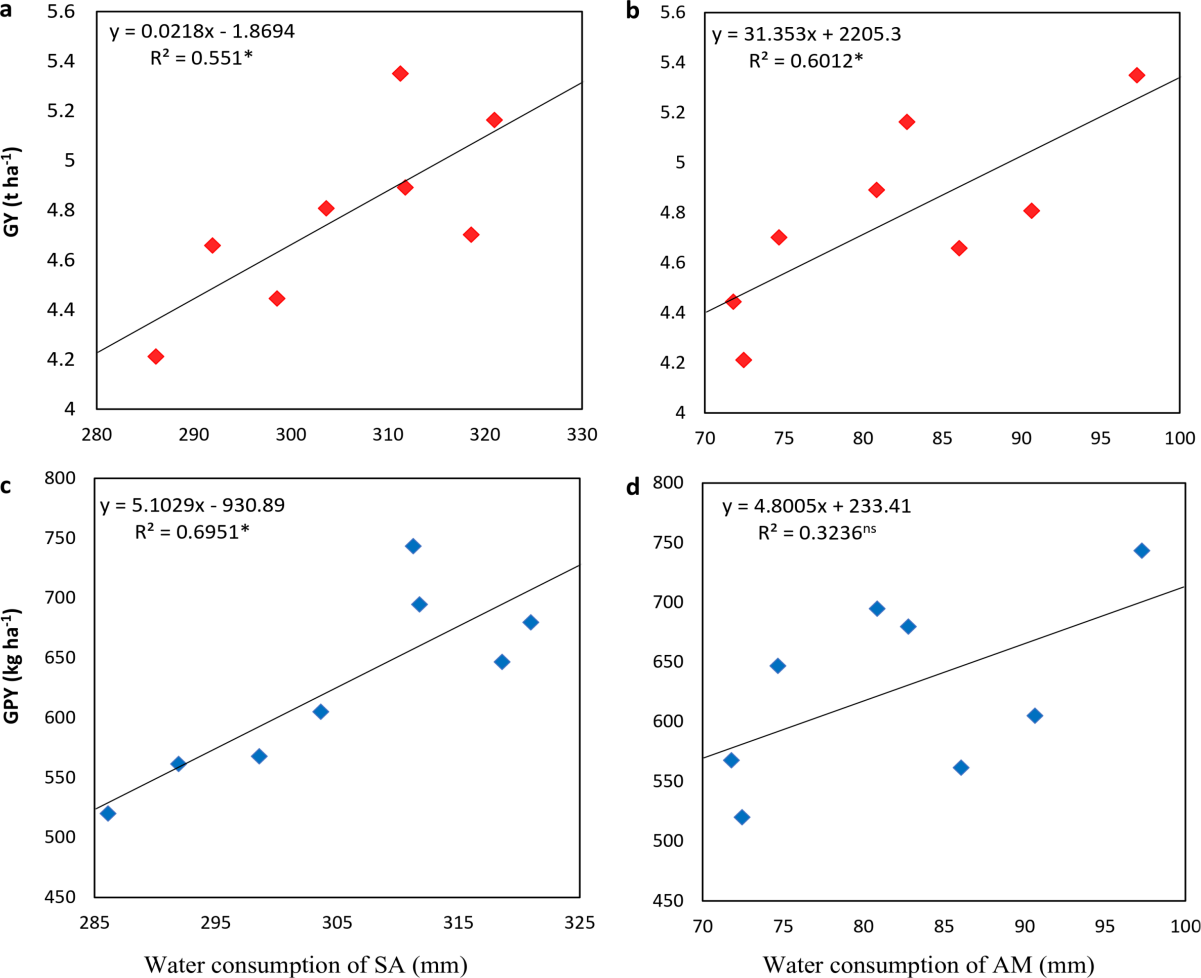
Relationship of grain yield (A, B) and grain protein yield (B, C) with soil water consumption at sowing to anthesis (SA) and anthesis to maturity (AM) of winter wheat. GY, grain yield; GPY, grain protein yield.

**Figure 8:**
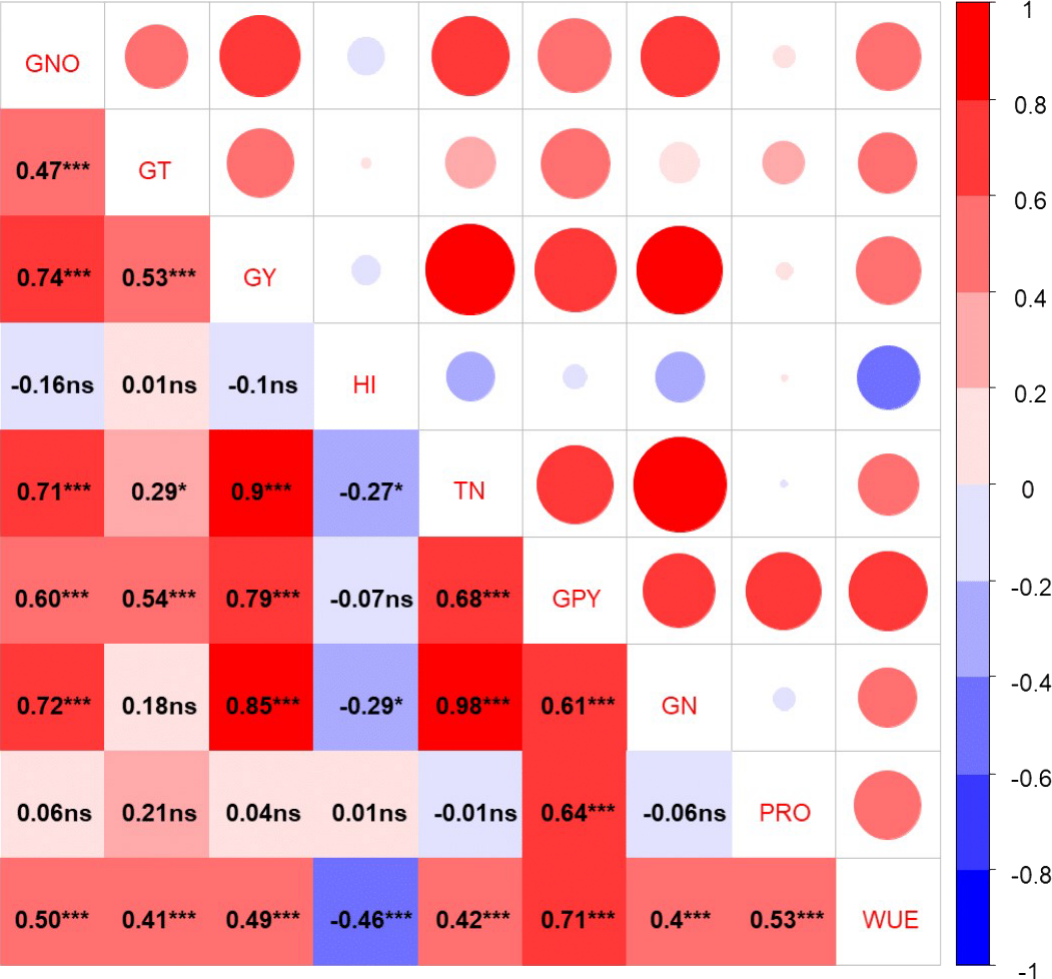
Pearson correlation coefficients among grain yield, protein yield and *N* conten.t. GNO, grain no.; GT, 1,000 grain weight; GY, grain yield; HI, harvest index, TN, total plant nitrogen; GPY, grain protein yield; GN, grain nitrogen; PRO, protein; ns, non-significant; *, **, ***, significant at 0.05, 0.01 and 0.001 probability level, respectively.

The grain yield was significantly and positively linked with soil water consumption from sowing to anthesis and anthesis to maturity (Fig. 7). Furthermore, grain protein yield was also increased by increasing the water consumption from sowing to anthesis with a fitted equation *y* = 5.1029x − 930.89, *R*^2^ = 0.695. Whereas, the relationship of grain protein yield with water consumption from anthesis to maturity was not significant. Grain yield was significantly and positively related to grain number, grain 1,000 weight, total plant N, and WUE (Fig. 8). Conversely, HI was significantly and negatively correlated with the total plant N, grain N, and WUE. Grain protein content was positively related to grain protein yield.

## Discussion

Wheat yield in semiarid dryland areas is highly affected by the variation in the amount and distribution of seasonal precipitation (Wang & Shangguan, 2015). Precipitation is important meteorological factor which affect soil water content. In Loess Plateau and other dryland areas, the soil water content at time of sowing is important for early growth of wheat and highly dependent on the precipitation during fallow season of dryland wheat (Kang et al., 2002; Rossato et al., 2017).

The wheat yield is linearly related to the soil water content (Musick et al., 1994; Qin, Chi & Oenema, 2013). In our study, soil water storage at sowing and anthesis was highest in the 2014–2015 which received the highest precipitation. The mean highest yield (5.11 t ha^−1^) was obtained in the year 2014-2015 which received the highest precipitation whereas, the lowest yield (4.45 t ha^−1^) was in 2015–2016 which received the lowest precipitation. This indicated that the soil water content is important for determining dryland wheat yield while water stress is limiting wheat yield at Loess Plateau. Some previous studies also showed that the higher water content at planting results in higher water consumption by the crop (Kang et al., 2002; Qin, Chi & Oenema, 2013). Our study indicated that the difference in soil water storage is due to variable rainfall during the fallow period and growth period of wheat. The lowest yield in 2015–2016 might be attributed to the less water content in 0–300 cm soil profile at anthesis. The variation in grain yield with years could be attributed to the difference in precipitation rates and precipitation pattern (Hemmat & Eskandari, 2006). Previous studies reported that the distribution pattern of rainfall, especially in soils with low water storage capacity may be more important than the total amount of rainfall in determining crop yield in dryland areas (Brunel, Seguel & Acevedo, 2013). Particularly water deficit during the reproduction and grain filling stages is severely limiting wheat production in dryland areas of Mediterranean climate mainly due to an unfavorable rainfall regime (Brunel, Seguel & Acevedo, 2013). In the Mediterranean climate, less than 30% of rainfall occurs during fallow. In contrast, in dryland Loess Plateau almost 60–70% of rainfall is concentrated in the fallow season but the later reproductive stages are less prone to water deficiency due to cool weather. Therefore, water storage during the fallow season matters more for determining yield in dryland Loess Plateau (Zhang et al., 2008; Wang & Shangguan, 2015). Some previous research also indicated that water stored at sowing time may be an important complement to the seasonal rains in dryland areas since this stored water may be more effective in promoting yield (Sun et al., 2010).

Results indicated that the grain yield of deep tilled fields was higher than no-tillage. The significant contribution of deep tillage to yield can be explained by greater water and N availability compared with no-tillage (Sarker et al., 2017). The soil water storage of 0–300 cm soil profile at sowing was higher with the DT than at NT but the effect was less pronounced in the year (2015–2016) with high precipitation at sowing and anthesis in 2015–2016. Adequate soil moisture is beneficial for the growth and development of rain-fed wheat, which directly affects the yield (Liang et al., 2019). Wheat yield found to be linearly related to the soil water content at planting (Musick et al., 1994). Present results showed higher soil water consumption after DT as compared to NT, which in turn increased the yield as grain yield is found to be positively related with the soil water consumption during growth stages of wheat. Increased in grain yield might also be attributable to high N uptake due to high soil water storage and N availability by tillage. Tillage during the fallow period improved the contribution of N translocation to grain (Liang et al., 2019) which in turn increased the seed yield (Sarker et al., 2017). Total aboveground dry biomass of wheat was higher after DT as compared to NT at the wintering stage and the same trend was observed until maturity. After the tillage practice during fallow time, plants mostly showed early rapid growth due to improved drainage by breaking hardpans as the soil absorbs more fallow season rainfall and high N availability is good to sustain early plant growth (Sarker et al., 2017).

WUE is closely related to the effectiveness of the use of precipitation in the dryland system, since rain is the sole source of water in drylands (Hatfield, Sauer & Prueger, 2001). Present study indicated that the WUE was affected by tillage and N rates. Deep tillage improved the WUE from 12.4 kg ha^−1^ mm^−1^ at NT to 13 kg ha^−1^ mm^−1^ under DT. A higher WUE indicates that the wheat plants can produce a higher yield by using less water and tillage improves WUE by increasing the available soil water content at sowing (Wang & Shangguan, 2015).

Soil tillage practices influence the physico-chemical characteristics of soil by altering carbon sequestration and nutrient distribution by incorporating crop residues and mineral or organic fertilizers (Neugschwandtner et al., 2014). In the present study, the deep tillage had increased the organic carbon, available N while reduced the soil bulk density, which were beneficial factors for improving soil quality and WUE. Tillage done at appropriate soil moisture level loosens the soil which increases the aeration and porosity and incorporate residues into deeper organic poor layers of soil (Dam et al., 2005; Osunbitan, Oyedele & Adekalu, 2005).

In present study, the DT had increased the total plant N uptake and N content in different plant parts as compared to NT. Increased N accumulation may be mainly attributable to increased soil N availability to plants by tillage (Soon, Brandt & Malhi, 2006; Ruisi et al., 2016). Ruisi et al. (2016) reported that, the N immobilization and N losses are higher under no-tillage system which cause reduction in soil mineral N whereas increase N losses. Organic N mineralization rate is often higher in plowed system than under no-tillage. Furthermore, the crop residues left on soil surface in no tillage condition increases N immobilization and might also be a factor for reducing soil N availability as compared to tillage (Giller et al., 2009). In loess plateau, most of the precipitation (>50%) occurs in summer (July to September) and pre-sowing availability of the water is the most important factor for N availability (Fu et al., 2014). According to Jan & Amanullah Hussain (2016), the higher yield of wheat could be achieved by adjusting the N fertilizer rate according to the precipitation rate.

The accumulation of N in plant parts was significantly affected by the N rates and tillage. N content in all plant parts was increased with increasing the application rate of N from 90 kg ha^−1^to 150 kg ha^−1^ and decreased or unaffected by further increasing N rate. This might be due to a higher utilization efficiency of N at 150 kg ha^−1^. Overall, maximum NUE was found under DT along with 150 kg ha^−1^ and minimum at NT along with 210 kg ha^−1^. These results indicated that tillage enhanced the availability, efficiency, and uptake of N as compared to NT. Tillage improved the transfer of N from soil to aboveground plant parts (Sarker et al., 2017). Ma et al. (2019) recommended that in order to balance NUE, grain yield and to reduce N loss the N application rate should be in the range of 120–171 kg ha^−1^.

Present results indicated that the soil water storage at anthesis and maturity was decreased with increasing N rate and a more significant decrease was observed in the year with high precipitation. Soil water consumption was enhanced by increasing the N rate and effect was more significant for soil water consumption from sowing to anthesis as compared to anthesis to maturity. Maximum water consumption at sowing to anthesis was recorded at 210 kg N ha^−1^ and anthesis to maturity at 150 kg N ha^−1^. In the present study, the increase in water consumption was explained by the enhanced plant growth by N application and higher soil water storage at sowing under deep tillage. Similarly, He et al. (2016) and Li et al. (2004) reported that soil water consumption was increased by increasing plant growth owing to higher soil water at sowing under different soil management practices. This indicate that the excessive use of N fertilizer makes plants to consume more soil water and causes the soil desiccation of high-yielding winter wheat fields of rainfed dryland areas (Li et al., 2004; He et al., 2016).

Grain protein content was increased linearly by the application of N from 0 to 150 kg ha^−1^ anddecreased at highest concentration of N. Increase in protein content by N application was higher under NT as compared to DT, which might be due to the greater crop response to N fertilization under the condition of low soil N availability (Rial-Lovera et al., 2016). A positive correlation was also determined between N content of soil and total protein content in soybean grain (Spoljar et al., 2009). Agronomic practices need to ensure optimal N fertilization is conducted when the plant can still incorporate the N into its grain and does not limit NUE. The protein change in response to N and late-season application time of N is generally greater and more reliable under irrigation than dryland production, because the N is usually incorporated with irrigation, increasing N uptake (Jones & Olson-Rutz, 2012). Whereas, under dryland conditions of Loess Plateau, N is mostly applied at once before sowing (Khan et al., 2017). The lower response of higher N applications in dryland production is likely because there is low chance of adequate rainfall to push N into the soil and promote N uptake (Sarker et al., 2017). Abedi, Alemzadeh & Kazemeini (2011) also indicated that the over application of N decreased the grain protein content.

Harvest index (HI) indicates the allocation of biomass to grain and partitioning between straw production and grain. Harvest index of modern wheat mostly ranges from 0.3 to 0.6. HI is determined by the genetic variations however, also influenced by environmental factors within certain climate region (Dai et al., 2016). In our study, HI was affected by the year, tillage and N application. Overall, HI in 2015–2016 was the highest (0.38) amongst all years which might be due to the variation in the pattern of rainfall. In 2015–2016, there was high rainfall from anthesis to maturity stage which might favor allocation of assimilates to grain yield whereas less dry matter was accumulated in plant vegetative tissues due to less rainfall in sowing-anthesis stage. Overall, the highest HI was recorded at 210 kg N ha^−1^ which indicated that by increasing N rate, increase in allocation of assimilates to grain yield was higher than accumulation in plant vegetative parts. This increase might be due to the effect of N rates on grain and biological yields, as suggested by Ghadikolayi, Kazemeini & Bahrani (2015), who reported that the highest HI of wheat was obtained at highest used N rate (180 kg ha^−1^).

## Conclusions

Our findings highlight that practicing deep tillage during fallow improved the soil water storage in 0–300 cm depth which increased water consumption during anthesis and maturity stages as compared to no-tillage. However, the effect of tillage was less prominent in year with high precipitation at sowing. Furthermore, grain yield was found to be positively related with the soil water consumption during growth stages of wheat. The higher yield under DT was due to higher N allocation in leaf, stem and grains and N use efficiency of wheat by changing soil properties and increasing N uptake. Application of 150 kg N ha^−1^ gave significantly higher N contents in plant parts and water use efficiency than other N rates. This might be due to a higher utilization efficiency of N at 150 kg ha^−1^. The grain yield, grain protein content and N contents in plant parts were not further increased by increasing N rate to 210 kg ha^−1^. Therefore, excessive N rates should not be effective under dryland conditions of Loess Plateau due to excessive plant growth and higher water consumption.

##  Supplemental Information

10.7717/peerj.8892/supp-1 Data S1 Raw data applied for data analysis

## Additional Information and Declarations

### Data Availability

The following information was supplied regarding data availability: All raw data are available as a Supplemental File.
